# The state of the art in organizational cognitive neuroscience: the therapeutic gap and possible implications for clinical practice

**DOI:** 10.3389/fnhum.2013.00808

**Published:** 2013-12-05

**Authors:** Carl Senior, Nick Lee

**Affiliations:** ^1^School of Life and Health Sciences, Aston UniversityBirmingham, UK; ^2^Aston Business School, Aston UniversityBirmingham, UK

**Keywords:** organizational psychology, clinical practice, neuroeconomics, neuromarketing, neuroscience methods

## Abstract

In the last decade, researchers in the social sciences have increasingly adopted neuroscientific techniques, with the consequent rise of research inspired by neuroscience in disciplines such as economics, marketing, decision sciences, and leadership. In 2007, we introduced the term organizational cognitive neuroscience (OCN), in an attempt to clearly demarcate research carried out in these many areas, and provide an overarching paradigm for research utilizing cognitive neuroscientific methods, theories, and concepts, within the organizational and business research fields. Here we will revisit and further refine the OCN paradigm, and define an approach where we feel the marriage of organizational theory and neuroscience will return even greater dividends in the future and that is within the field of clinical practice.

## Introduction

In this article the clinical concept of the “therapeutic gap” is discussed in light of the “research-practice gap” central to ongoing debate within business research. Inspired by Grimshaw et al. (2012), we explore two key questions; what types of business research could offer clinical insights, and how? What are the pitfalls such research may encounter, with particular regard to translational practices and public understanding? Such issues are vital in light of the ever-increasing public interest in translational neuroscience, and especially in the findings of such research as applied to our own lives as employees and consumers (see e.g., O’Connor et al., 2012). In partial answer to the first question, we suggest that certain types of organizational cognitive neuroscience (OCN; Senior et al., 2011) could provide a beneficial context in which to conduct research with direct therapeutic implications. In doing so, we show that taking account of such issues should also lead to better science, and provide examples of research that could be of use in this regard. Throughout, we address the second question by pointing out significant concerns with certain existing approaches to such research. Finally, we conclude with a call to arms for researchers to consider the therapeutic implications of their work, further drawing neuroscientific and business researchers closer, ultimately leading to research with greater translational potential.

## Organizational cognitive neuroscience and the therapeutic gap

Many scientists feel that the primary focus of their endeavors should be on ensuring the scientific rigor of their methods, and the ensuing creation of knowledge. While these are laudable goals, knowledge translation can sometimes be overlooked. Yet, the communication of any findings to the wider community of relevance, in order that it be put into practice, is a vital part of the wider role of science (Plebani and Marincola, 2006). Further, in contemporary scientific environments, where scientists often have to compete with other funding priorities, superior knowledge translation can be of key importance in securing ongoing research streams.

Across the social sciences, a common frustration concerns the distance between the research activities carried out within laboratories, and their subsequent practical application in the real world (Cousins and Simon, 1996). This has been referred to within the clinical sciences as the *therapeutic gap*, and is generally regarded as being the fundamental barrier to developing a comprehensive model of health behavior (Bero et al., 1998; Grimshaw et al., 2012). Importantly, the therapeutic gap is not merely an artefact of the publication delay so commonly criticized by scientists. Rather, it is the gap between scientific publication of research findings and their subsequent *application* towards the development of evidence-based practice.

There have been several recent attempts to close the therapeutic gap in our understanding of a range of diseases from a variety of avenues—and perhaps the most surprising of these avenues is from marketing science (Javor et al., 2013). In light of such recent contributions, the utility of the emerging field of OCN is discussed here as a framework for understanding how research findings from the laboratory can be translated to clinical benefits. OCN is an umbrella term that encompasses a variety of subordinate domains, therefore the aforementioned neuromarketing as well as neuroeconomics will be discussed further.

Beginning with neuroeconomics, this field has probably seen the most prolific advancement in its approach to the translation of knowledge across a range of different domains (Clithero et al., 2008). It is a field where various outputs do indeed have direct implications across a rich and diverse constituency (Levallois et al., 2012). It is also clear that such diversity has immense potential to straddle different scientific disciplines and in doing so connect distinct research networks, resulting in genuinely innovative research collaborations (Christopoulos et al., 2009).

The generation of such diverse research collaborations has seen the development of a “trade language that facilitates communication between disparate cultures” (Levallois et al., 2012, p. 8). This common language, spanning several research areas and laboratories, is facilitating the closure of the gap between research and practice and in turn ensuring the rapid translation of research findings from the laboratory into real world practice (Kishida, 2012). In fact, even though most neuroeconomic research is constrained by the traditional limitations of the laboratory (Kuhnen and Knutson, 2005) such research has made significant discoveries with direct clinical relevance.

One very notable example is seen with the work by Hackman and colleagues, who uncovered a significant link between parental socio-economic status and the subsequent neurocognitive development of their offspring (Hackman and Farah, 2009; Hackman et al., 2010). Here there is very clear link between parental economic position and the health of family members at a cortical level of analysis—a link that would have obvious implications for subsequent clinical ontology. In this regard, the study of economic theory has a direct role in understanding the cognitive development of children. Thus, *ceteris paribus*, the application of economic theory to improve the lives of the parents could improve the cognitive development of their children. Here, one could argue that the therapeutic gap has been reduced to a significant degree—or even closed completely.

Beyond this, one can also observe the continuous generation of new hypotheses, further elucidating areas of clinical interest. Take recent work in the field of neuropsychiatry and the application of computational theory to model psychiatric phenomena for example (Montague et al., 2011). This approach is based on the fundamentals of modeling economic exchange, and promises to be a fertile area in the future with much clinical potential to enhance the testing and approval of new drugs. Similarly, Hasler (2012) has further developed the clinical potential of neuroeconomics, describing the possible benefits of clinical interventions based on the application of game theory (Hasler, 2012). Abnormal social behavior can be formalized using behavioral game theory. As such Psychiatry will benefit from neuroeconomics’ unified theory of human behavior (Glimcher and Rustichini, 2004). Ultimately such approaches will ensure that neuroeconomics will have great clinical utility, helping to further narrow the therapeutic gap in the years to come (Bernheim, 2009).

A second approach born from the OCN paradigm that promises to help close the therapeutic gap is the emerging field of neuromarketing (Lee et al., 2007). The steady evolution of this specific approach is of particular importance in understanding the therapeutic gap, because at its core neuromarketing is essentially the application of neuroscience to understand decision-making within a market context. Such fundamental market behaviors are manifest throughout our entire repertoire of social behaviors and address core questions regarding the human condition (Fisher et al., 2010; Ouazzani Touhami et al., 2011). Thus, it is quite easily conceivable that neuromarketing research may help to alleviate a range of social pathologies (Montague et al., 2002; Babiloni et al., 2007; Dumas et al., 2010; Babiloni and Astolfi, 2012; Tikka et al., 2012). That said, while, reports in this regard have been published in some of the most prestigious journals in the world (The Lancet Neurology, 2004; Ariely and Berns, 2010), we would urge at this juncture that more effort is needed before the true utility of neuromarketing can be seen in this regard.

In fact, some would argue that it is neuromarketing itself that has driven the onset of various pathologies such as compulsive shopping and childhood obesity (Fisher et al., 2010; Jain, 2010), even going so far as to say that “neuromarketing research is also allowing advertisers to appeal to the subconscious to which children may be particularly vulnerable” (Jain, 2010, p. 425). Of course, we agree completely that pediatric (commercial) neuromarketing endeavors are simply untenable (Murphy et al., 2008), and it is with the study of adult populations that we will see neuromarketing science significantly close the therapeutic gap.

However, this will see neuromarketing researchers embracing a Faustian pact of sorts with critics. In particular, with the identification of a discrete region in the human brain that is activated for consumer behavior, it may be noted that we are implicitly accepting the well-established fallacy of a “buy button” in the brain (See Blakeslee, 2004). This though is something of a gross over simplification, even though it should be acknowledged that the various social processes that mediate financial exchange for goods are starting to be mapped out. Such processes would be viable candidates for therapeutic intervention, as long as they are considered within the constraints of their cognitive domain. Here we are assuming that any given brain area activated for a particular social task must be involved in one (or many) subordinate tasks that may drive the overarching social behavior.

While the complex processes that we undergo during a purchase decision are beginning to be broken down and mapped to various brain areas, we are as yet unable to show the specificity of any area to any individual behaviors. Yet, by delineating the social processes that mediate purchase exchanges, and mapping each of these processes to a different network, it is possible to interrogate cortical systems to identify how and why they are involved in our daily purchase decisions. Only once such networks have been identified can we then start to examine their role in pathological states such as compulsive gambling or shopping addiction (Aboujaoude et al., 2003).

While the simplistic belief in a “buy button” in the brain has been widely ridiculed (e.g., http://www.russpoldrack.org/2011/10/nyt-editorial-fmri-complete-crap.html), it is unavoidable that the cognitive architecture that drives purchase behavior must indeed exist in some form. A more ecologically-valid way of conceptualizing this architecture is to consider the complex interactions that occur at neural network level during the various social processes that occur during market exchanges. From a neuroscientific perspective this is standard practice (see e.g., Friston, 2000), and we have previously urged scholars within the organizational sciences to adopt such an approach (Lee et al., 2012). It is only with such a forward inferential model that insights into compulsive pathology can be made (van Holst et al., 2012). Here there is indeed great potential to close the therapeutic gap in relation to impulse control disorders that occur within the market (Aboujaoude et al., 2003; Black, 2007).

In a translational context, it is not enough to simply conduct scientifically-rigorous research. Rather, the interpretation and use of such research by non-scientists (such as clinical practitioners and the general public) must be taken into account. Within a neuroscience context, the inherent technical, philosophical, and conceptual complexity places an even greater responsibility on scientists in this regard. As such, complimenting the contributions of neuroeconomics and neuromarketing is the recent grassroots initiative within the neuroscience research fraternity to educate its readers (Illes et al., 2010). For example, one can now expect, as a matter of course, passionate rhetoric in response to research reports that have failed to encapsulate a full description of the limitations of the application of technologies such as fMRI for example (Vul et al., 2009).^1^

The drive to empower a greater public (and by extension practitioner) understanding of the brain has evolved from neuroethics (Roskies, 2002; Farah, 2012; Shadlen and Roskies, 2012). A particular catalyst of these efforts is the finding that brain scan images may have a persuasive effect on the reader (See also McCabe and Castel, 2008; Farah and Hook, 2013). The significance of such an effect has led to scholars calling for limits on the use of brain scan imagery in areas such as the judiciary and national security (Senior, 2008; Rippon and Senior, 2010), and has also seen calls to develop regulatory processes that protect the various participants when interacting with such technology (Garnett et al., 2011; Farah, 2012). This has important implications with regards to the therapeutic gap in clinical practice. Specifically, the rise of such educational initiatives for both neuroscientists (Sahakian and Morein-Zamir, 2009; Kehagia et al., 2012) and the general public alike, will inevitably serve to develop a framework of normative neuroscience that clearly describes the extent to which any findings can be extrapolated to the real world, including clinical practice.

One example is the recent attempts to change the way that neuroscientific research is portrayed in non-academic reports. This work has revealed that neuroscientific research is afforded “substantial and authoritative weight” (p.725) on the development of governmental policy (Racine et al., 2010). In addition to this, research has also explored the ways that neuroscientific research is portrayed within the pages of mainstream newspapers (Rose, 2007). Indeed, in a recent study on neuroscientific reporting in the public sphere, O’Connor et al. (2012) found that neuroscience research was reported within a small number of thematic contexts—such as brain optimization or futuristic phenomena—and that this grouping was in fact a significant factor in the effective facilitation of the transfer of knowledge from the research laboratory to the real world (O’Connor et al., 2012). The authors are unequivocal in stating the importance of fully understanding the limitations of neuroscientific research and how they effect applications in the real-world; “*neuroscience does not take part in a vacuum, and it is important to maintain sensitivity to the social implications, whether positive or negative, it may have as it manifests in real-world contexts. It appears that the brain has been instantiated as a benchmark in public dialogue, and a reference to brain research is now a powerful rhetorical tool”* (p. 225).

Thus it would seem that the neuroscience community itself is taking the vanguard with regard to closing the therapeutic gap, and ensuring that any application of neuroscience research is embedded within the realistic confines of its applicability. However it remains to be seen whether future scientific results from the neuroscience research community will result in a full transfer of knowledge into the public domain, and whether OCN as a context will facilitate this transfer. It is the formation of a common trade language that spans across areas such organizational behavior, marketing, and clinical therapy that will return the greatest dividends in closing the therapeutic gap.

## Conclusions

Our interest here was to explore the idea that OCN research could help drive improvements in clinical practice, helping to close the therapeutic gap. Interactions and experiences in relation to organizations, such as within the workplace, or the market, provide much of what it means to be human in contemporary society. It seems eminently logical that we begin to consider whether investigation of our behavior within these contexts can bear fruit in terms of improving the human condition. Certainly, authors have not been shy in blaming contemporary human problems on modern work, marketing, or media, and therefore it stands to reason that investigations of these phenomena may be important directions in solving contemporary human pathology.

As can be seen from the discussion above the translation of knowledge from the organizational sciences to address the therapeutic gap is currently underway. However, to facilitate such translation further we would urge researchers to consider five questions that have been designed to expedite knowledge translation across various disciplines (Grimshaw et al., 2012). What information should be transferred? To whom should it be transferred? By whom? How? And with what effect? Taken together these questions provide an organizing framework for a highly effective knowledge transfer strategy and one that should see research in the organizational fields making an impact within the clinical domain (Lavis et al., 2003).

However, while we urge researchers in OCN-related fields to consider the therapeutic implications of their work, we also urge caution. In the move to embrace techniques such as fMRI, it is easy to over-interpret findings and draw erroneous, and potentially harmful, conclusions. This is particularly relevant within organizational research contexts, where readers and users of research may have less experience and knowledge of how to correctly interpret neuroscience findings. As always, we urge researchers to form collaborative teams, including experts in all fields of relevance. Such endeavors are vital when attempting to draw clinical conclusions from OCN studies. Even so, we hope researchers will take up this call to arms, and explore the clinical potential of their work. In doing so, we envisage significant advances being made towards the understanding and treatment of many of the pathologies that plague our society.

## Conflict of interest statement

The authors declare that the research was conducted in the absence of any commercial or financial relationships that could be construed as a potential conflict of interest.

## Footnotes

^1^The neuroscience community is prolific in this regard and has ensured that various caveats enter the public domain in high profile mainstream publications as well see e.g., http://www.nytimes.com/2007/11/14/opinion/lweb14brain.html and http://www.russpoldrack.org/2011/10/nyt-letter-to-editor-uncut-version.html

## References

[B1] Aboujaoude E. Gamel N. Koran L. (2003). A 1-year naturalistic follow-up of patients with compulsive shopping disorder. J. Clin. Psychiatry 64, 946–950 10.4088/jcp.v64n0814 12927011

[B2] Ariely D. Berns G. S. (2010). Neuromarketing: the hope and hype of neuroimaging in business. Nat. Rev. Neurosci. 11, 284–292 10.1038/nrn2795 20197790 PMC2875927

[B3] Babiloni F. Astolfi L. (2012). Social neuroscience and hyperscanning techniques: past, present and future. Neurosci. Biobehav. Rev. [Epub ahead of print]. 10.1016/j.neubiorev.2012.07.006 22917915 PMC3522775

[B4] Babiloni F. Cincotti F. Mattia D. De Vico Fallani F. Tocci A. Bianchi L. (2007). High resolution EEG hyperscanning during a card game. Conf. Proc. IEEE Eng. Med. Biol. Soc. 2007, 4957–4960 10.1109/iembs.2007.4353453 18003119

[B5] Bernheim B. (2009). On the potential of neuroeconomics: a critical (but hopeful) appraisal. Am. Econ. J. Microeconomics 1, 1–41 10.1257/mic.1.2.1

[B6] Bero L. A. Grilli R. Grimshaw J. M. Harvey E. Oxman A. D. Thomson M. A. (1998). Closing the gap between research and practice: an overview of systematic reviews of interventions to promote the implementation of research findings. The cochrane effective practice and organization of care review group. BMJ 317, 465–468 10.1136/bmj.317.7156.465 9703533 PMC1113716

[B7] Black D. (2007). A review of compulsive buying disorder. World Psychiatry 6, 14–18 17342214 PMC1805733

[B52] Blakeslee S. (2004). If your brain has a ‘buy button’ what pushes it? The New York Times. October 19th.

[B8] Christopoulos G. Tobler P. Bossaerts P. Dolan R. Schultz W. (2009). Neural correlates of value, risk, and risk aversion contributing to decision making under risk. J. Neurosci. 29, 12574–12583 10.1523/jneurosci.2614-09.2009 19812332 PMC2794196

[B9] Clithero J. Tankersley D. Huettel S. (2008). Foundations of neuroeconomics: from philosphy to practice. PLoS Biol. 6:e298 10.1371/journal.pbio.0060298 19067493 PMC2586372

[B10] Cousins J. Simon M. (1996). The nature and impact of policy induced partnerships between research and practice communities. Educ. Eval. Policy Anal. 18, 199–218 10.3102/01623737018003199

[B11] Dumas G. Nadel J. Soussignan R. Martinerie J. Garnero L. (2010). Inter-brain synchronization during social interaction. PLoS One 5:e12166 10.1371/journal.pone.0012166 20808907 PMC2923151

[B13] Farah M. (2012). Neuroethics: the ethical, legal, and societal impact of neuroscience. Annu. Rev. Psychol. 63, 571–591 10.1146/annurev.psych.093008.100438 19575613

[B14] Farah M. Hook C. (2013). The seductive allure of seductive allure. Perspect. Psychol. Sci. 8, 88–90 10.1177/1745691612469035 26172255

[B15] Fisher C. E. Chin L. Klitzman R. (2010). Defining neuromarketing: practices and professional challenges. Harv. Rev. Psychiatry. 18, 230–237 10.3109/10673229.2010.496623 20597593 PMC3152487

[B16] Friston K. (2000). The labile brain. I. Neuronal transients and non linear coupling. Philos. Trans. R. Soc. Lon. B Biol. Sci. 355, 215–236 10.1098/rstb.2000.0560 10724457 PMC1692735

[B17] Garnett A. Whiteley L. Piwowar H. Rasmussen E. Illes J. (2011). Neuroethics and fMRI: mapping a fledgling relationship. PLoS One 6:e18537 10.1371/journal.pone.0018537 21526115 PMC3081297

[B51] Glimcher P. W. Rustichini A. (2004). Neuroeconomics: the consilience of brain and decision. Science 15, 447–452 10.1126/science.1102566 15486291

[B18] Grimshaw J. Eccles M. Lavis J. Hill S. Squires J. (2012). Knowledge translation of research findings. Implement. Sci. 7, 1–29 10.1186/1748-5908-7-50 PMC3462671 22651257

[B19] Hackman D. A. Farah M. J. (2009). Socioeconomic status and the developing brain. Trends Cogn. Sci. 13, 65–73 10.1016/j.tics.2008.11.003 19135405 PMC3575682

[B20] Hackman D. A. Farah M. J. Meaney M. J. (2010). Socioeconomic status and the brain: mechanistic insights from human and animal research. Nat. Rev. Neurosci. 11, 651–659 10.1038/nrn2897 20725096 PMC2950073

[B21] Hasler G. (2012). Can the neuroeconomics revolution revolutionise psychiatry? Neurosci. Biobehav. Rev. 36, 64–78 10.1016/j.neubiorev.2011.04.011 21550365

[B22] Illes J. Moser M. A. McCormick J. B. Racine E. Blakeslee S. Caplan A. (2010). Neurotalk: improving the communication of neuroscience research. Nat. Rev. Neurosci. 11, 61–69 10.1038/nrn2773 19953102 PMC2818800

[B24] Jain A. (2010). Temptations in cyberspace: new battlefields in childhood obesity. Health Aff. (Millwood) 29, 425–429 10.1377/hlthaff.2010.0107 20194983

[B25] Javor A. Koller M. Lee N. Chamberlain L. Ransmayr G. (2013). Neuromarketing and consumer neuroscience: contributions to neurology. BMC Neurol. 13:13 10.1186/1471-2377-13-13 23383650 PMC3626833

[B26] Kehagia A. A. Tairyan K. Federico C. Glover G. H. Illes J. (2012). More education, less administration: reflections of neuroimagers’ attitudes to ethics through the qualitative looking glass. Sci. Eng. Ethics. 18, 775–788 10.1007/s11948-011-9282-2 21626219

[B27] Kishida K. (2012). A computational approach to “free will” constrained by the games we play. Front. Integr. Neurosci. 6:85 10.3389/fnint.2012.00085 23060761 PMC3459012

[B28] Kuhnen C. M. Knutson B. (2005). The neural basis of financial risk taking. Neuron 47, 763–770 10.1016/j.neuron.2005.08.008 16129404

[B29] Lavis J. Robertson D. Woodside J. McLeod C. Abelson J. (2003). How can research organisations more effectively transfer research knowledge to decision makers? Milbank Q. 81, 221–222 12841049 10.1111/1468-0009.t01-1-00052 PMC2690219

[B30] Lee N. Broderick A. Chamberlain L. (2007). What is “neuromarketing”? A discussion and agenda for future research. Int. J. Psychophysiol. 63, 199–204 10.1016/j.ijpsycho.2006.03.007 16769143

[B31] Lee N. Senior C. Butler M. (2012). The domain of organisational cognitive neuroscience: theoretical and empirical challenges. J. Manage. 38, 921–931 10.1177/0149206312439471

[B32] Levallois C. Clithero J. Wouters P. Smidts A. Huettel S. (2012). Translating upwards: linking the neural and social sciences via neuroeconomics. Nat. Rev. Neurosci. 13, 789–797 10.1038/nrn3354 23034481

[B33] McCabe D. Castel A. (2008). Seeing is believing: the effect of brain images on judgments of scientific reasoning. Cognition 107, 343–352 10.1016/j.cognition.2007.07.017 17803985

[B34] Montague P. Berns G. Cohen J. McClure S. Pagnoni G. Dhamala M. (2002). Hyperscanning: simultaneous fMRI during linked social interactions. Neuroimage 16, 1159–1164 10.1006/nimg.2002.1150 12202103

[B35] Montague P. Dolan R. Friston K. Dayan P. (2011). Computational psychiatry. Trends Cogn Sci. 16, 72–80 10.1016/j.tics.2011.11.018 22177032 PMC3556822

[B36] Murphy E. Illes J. Reiner P. (2008). Neuroethics neuromarketing. J. Consum. Behav. 7, 293–302 10.1002/cb.252

[B37] O’Connor C. Rees G. Joffe H. (2012). Neuroscience in the public sphere. Neuron 74, 220–226 10.1016/j.neuron.2012.04.004 22542177

[B38] Ouazzani Touhami Z. Benlafkih L. Jiddane M. Cherrah Y. El Malki H. O. Benomar A. (2011). Neuromarketing: when marketing meet neurosciences. Rev. Neurol. (Paris) 167, 135–140 10.1016/j.neurol.2010.07.025 20934735

[B39] Plebani M. Marincola F. M. (2006). Research translation: a new frontier for clinical laboratories. Clin. Chem. Lab. Med. 44, 1303–1312 10.1515/cclm.2006.238 17087640

[B40] Racine E. Waldman S. Rosenberg J. Illes J. (2010). Contemporary neuroscience in the media. Soc. Sci. Med. 71, 725–733 10.1016/j.socscimed.2010.05.017 20609506 PMC2925284

[B41] Rippon G. Senior C. (2010). Neuroscience has no role in national security. Am. J. Bioeth. 1, 37–38 10.1080/21507741003699322

[B42] Rose N. (2007). The Politics of Life Itself: Biomedicine, Power, and Subjectivity in 21st Century. Princeton, New Jersey: Princeton University Press

[B43] Roskies A. (2002). Neuroethics for the new millenium. Neuron 35, 21–23 10.1016/s0896-6273(02)00763-8 12123605

[B44] Sahakian B. Morein-Zamir S. (2009). Neuroscientists need neuroethics teaching. Science 325, 147 10.1126/science.325_147a 19589983

[B45] Senior C. (2008). The persuasive power of brain scan images. Am. J. Bioeth. 8, 60–61 10.1080/15265160802521021 19085482

[B46] Senior C. Lee N. Butler M. (2011). Organizational cognitive neuroscience. Organ. Sci. 22, 804–815 10.1287/orsc.1100.0532

[B47] Shadlen M. N. Roskies A. L. (2012). The neurobiology of decision-making and responsibility: reconciling mechanism and mindedness. Front. Neurosci. 6:56 10.3389/fnins.2012.00056 22536171 PMC3332233

[B12] The Lancet Neurology (2004). Neuromarketing: beyond branding. Lancet Neurol. 3, 71 10.1016/s1474-4422(03)00643-4 14746993

[B48] Tikka P. Aleksander V. de Borst A. Pugliese R. Ravaja N. Kaipainen M. (2012). Enactive cinema paves the way for understanding complex real time social interaction in neuroimaging experiments. Front. Hum. Neurosci. 6:298 10.3389/fnhum.2012.00298 23125829 PMC3485651

[B49] van Holst R. van der Meer J. McLaren D. van den Brink W. Goudriaan A. (2012). Interactions between affective and cognitive processing systems in problematic gamblers: a functional connectivity study. PLoS One 7:e49923 10.1371/journal.pone.0049923 23209619 PMC3509135

[B50] Vul E. Harris C. Winkielman P. Pashler H. (2009). Puzzingly high correlations in fMRI studies of emotion, personality and social cognition. Perspect. Psychol. Sci. 4, 274–290 10.1111/j.1745-6924.2009.01125.x 26158964

